# Synergistic Effects of The Enhancements to Mitochondrial ROS, p53 Activation and Apoptosis Generated by Aspartame and Potassium Sorbate in HepG2 Cells

**DOI:** 10.3390/molecules24030457

**Published:** 2019-01-28

**Authors:** Daofeng Qu, Mengxue Jiang, Dongping Huang, Hui Zhang, Lifang Feng, Yuewen Chen, Xuan Zhu, Suhua Wang, Jianzhong Han

**Affiliations:** 1 Food Safety Key Laboratory of Zhejiang Province, School of Food Science and Biotechnology, Zhejiang Gongshang University, Hangzhou 310035, China; daofeng@mail.zjgsu.edu.cn (D.Q.); 17826850974@163.com (M.J.); hdp2097@163.com (D.H.); zoechang224@gmail.com (H.Z.); fenglifang705@126.com (L.F.); chenyw@zjsu.edu.cn (Y.C.); zhuxuan@zjsu.edu.cn (X.Z.); 2 Wenzhou Entry-exit Inspection and Quarantine Bureau, Wenzhou 325027, China; 1410080129@pop.zjgsu.edu.cn

**Keywords:** food additives, HCA, joint toxicity, mitochondria pathway, p53, ROS

## Abstract

The safety of food additives has been widely concerned. Using single additives in the provisions of scope is safe, but the combination of additives, may induce additive, synergy, antagonism and other joint effects. This study investigated the cytotoxicity of aspartame (AT) together with potassium sorbate (PS). Thiazolyl Blue Tetrazolium Bromide (MTT) assay indicated that AT and PS had IC_50_ values of 0.48 g/L and 1.25 g/L at 24 h, respectively. High content analysis (HCA) showed that both AT and PS had a negative effect on mitochondrial membrane potential (MMP), reactive oxygen species (ROS) and DNA damage while the joint group behaved more obviously. The biochemical assays revealed typical cell morphological changes and the activation of cytochrome c and caspase-3 verified apoptosis induced by AT together with PS. With dissipation of MMP and increase of cell membrane permeability (CMP), it indicated AT together with PS-induced apoptosis was mediated by mitochondrial pathway. Meanwhile, p53 were involved in DNA damage, and the ratio of Bax/Bcl-2 was increased. Moreover, excessive ROS induced by AT together with PS is a key initiating factor for apoptosis. All these results proved that p53 was involved in apoptosis via mitochondria-mediated pathway and the process was regulated by ROS.

## 1. Introduction

Food additives, as a large category of compounds, are widely used in daily life, whose safety has been a widespread concern. The current evaluation on safety of food additives is based on a single species to formulate the appropriate maximum use. In fact, one kind of food may contain more than a dozen food additives, thus the daily intake of food additives via food for an individual might involve dozens or even hundreds of species. Single additives in the provisions of the scope of use are safe, but the combination of several additives may induce additive, synergistic, antagonistic and other joint effects [1]. Therefore, to explore and evaluate the joint toxicity of food additives for its safe usage is of great significance.

Potassium sorbate (PS) is the potassium salt of sorbic acid, mainly used as a food preservative. It is effective in a variety of applications, including food, wine and personal care products [2]. The acceptable daily intake (ADI) for human consumption is 25 mg/kg and for the average adult (70 kg) is 1750 mg daily [3]. Aspartame (AT) is an artificial, non-saccharide sweetener used as a sugar substitute in some foods and beverages [4]. The FAO/WHO Joint Committee of Food Additives (JECFA) and the European Commission Committee on Food Science have determined that the value of AT is 40 mg/kg body weight [5], while FDA has set its ADI for aspartame at 50 mg/kg.

In the past several years, High Content Analysis (HCA) technology based on fluorescence microscopy imaging has emerged as a kind of cell experimental detection system, which provides a new way to evaluate a variety of cell biological parameters including subcellular compartments, multicellular structures and model organisms concurrently [6]. HCA has already been demonstrated to be a valuable and promising tool for mechanistic understanding in the field of compound-induced hepatotoxicity under the premise of maintaining the structure and function of whole cells [7,8,9]. HCA provides us with a more convenient method to evaluate the joint effect of these compounds in foods compared with conventional methods, such as animal experiments or the marrow cell micronucleus test, which is inefficient and inconvenient. Human hepatocellular carcinoma (HepG2) cells show similar cellular functions to normal hepatocytes and have a high degree of morphological and functional differentiation [10]; therefore, HepG2 cell lines are usually selected as appropriate models for drug targeting and environmental chemical safety assessment [11].

In daily life, AT and PS are often used in many foods and are easily ingested at the same time. Currently, there is not much report on the combined effects of these two food additives, so the exploration in this area is necessary. The aim of this study was to determine the cytotoxicity of AT together with PS and to investigate the molecular mechanism and the possible pathway of apoptosis induced by AT together with PS.

## 2. Results

### 2.1. The Effect of AT and PS Inhibited the Proliferation in HepG2 Cells

**Figure 1:**
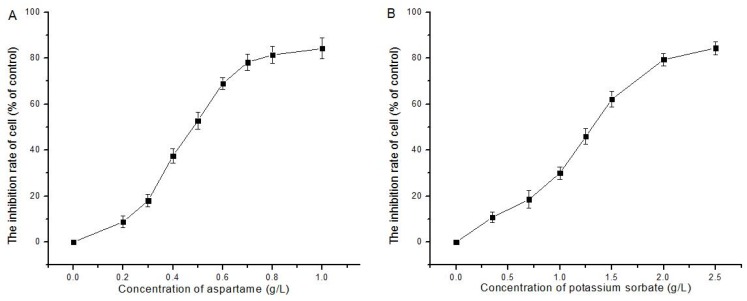
Effect of AT and PS on the proliferation of HepG2 cells: (**A**) the proliferation of HepG2 cells inhibited by the treatment of AT (0–1.0 g/L) for 24 h; and (**B**) the proliferation of HepG2 cell inhibited by the treatment of PS (0–2.5 g/L) for 24 h. The data are presented as mean ± SD.

The result in Figure 1 show that AT and PS were able to inhibit the proliferation of HepG2 cells in a dose-dependent manner with an IC_50_ of 0.48 g/L and 1.25 g/L, respectively, indicating AT inhibited HeoG2 cells proliferation more than PS did.

#### Apoptosis induced by AT together with PS in HepG2 cells

**Figure 2:**
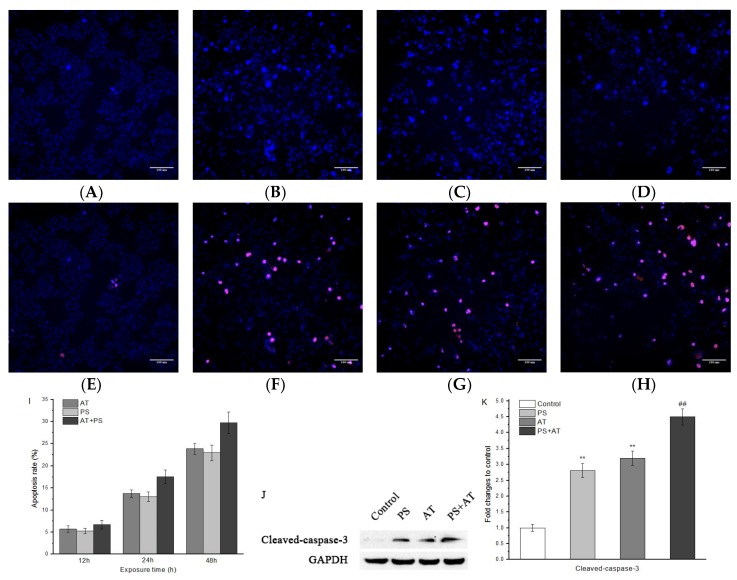
Effect of AT and PS on cellular morphology and apoptosis of HepG2 cells. The MD ImageXpress Micro XLS system was used to obtain images of HepG2 cells. (**A**–**D**) Effects of control group, AT group, PS group and joint group on cell nucleus of HepG2 cells for 24 h. HepG2 cells were stained with Hoechst 33342. (**E**–**H**) Effects of control group, AT group, PS group and joint group of HepG2 cells for 24 h. HepG2 cells were double-stained with Hoechst 33,342 and PI. Scale bars = 100 μm. (**I**) Effects of high dose AT group, PS group and joint group on apoptosis rate of HepG2 cells for 12, 24 and 48 h. (**J**) The expression of cleaved-caspase-3 in HepG2 cells. The cells were treated with high-dose separate PS or AT and joint group for 24 h. (**K**) Quantitation of cleaved-caspase-3 protein expression by densitometry, and the data were normalized using the GAPDH signal. The data are presented as mean ± SD. ** indicated *P* < 0.01, compared with the control group; ^##^ indicated *P* < 0.01, compared with AT and PS group.

As shown in Figure 2, AT- and PS-induced toxicity was indicated by HCA, which demonstrated an increase in cell shrinkage and the number of apoptotic cells. Cells were double-stained with Hoechst 33,342 for nuclei observation and with PI for apoptosis detection. Untreated HepG2 cells had regular contours and were round. When cells were exposed to joint groups for 24 h, they showed high PI signals. Meanwhile, the results obtained by Western blot show that both AT and PS could significantly increase the protein level of cleaved-caspase-3 and the increase of joint group was more obvious (Figure 2J,K).

Figure 2I shows that, after treatment with separate AT or PS and joint group for 12, 24, and 48 h, apoptosis rate increased in a dose-dependent manner to 5.72 ± 0.73%, 5.28 ± 0.59%, and 6.73 ± 0.97%; 13.81 ± 0.89%, 13.06 ± 1.07%, and 17.58 ± 1.54%; and 23.84 ± 1.24%, 22.92 ± 1.79%, and 29.73 ± 2.47%, respectively. These results prove that AT and PS inhibited cell proliferation by inducing cell apoptosis and the inhibitory effect of joint group was more pronounced.

### 2.2. AT Together with PS Induced the Changes of MMP, CMP and Protein Content of Cytochrome c in HepG2 Cells

**Figure 3:**
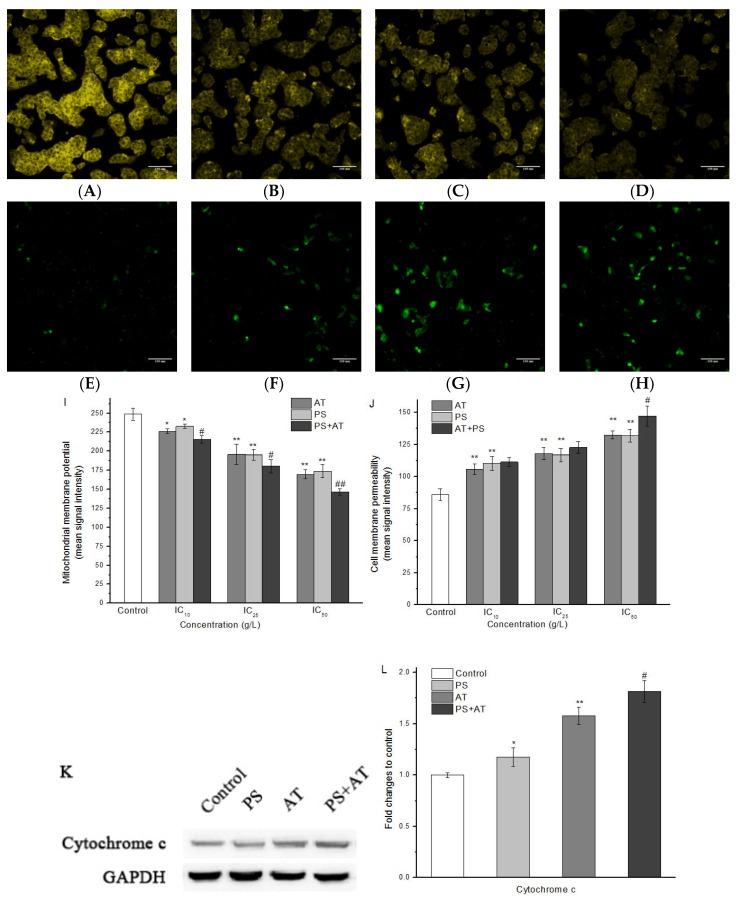
Imaging of MMP and CMP of HepG2 cells induced by AT and PS. The MD ImageXpress Micro XLS system was used to obtain images of HepG2 cells. (**A**–**D**) Effects of control group, AT group, PS group and joint group on MMP of HepG2 cells for 24 h. (**E**–**D**) Effects of control group, AT group, PS group and joint group on CMP of HepG2 cells for 24 h. Scale bars = 100 μm. (**I**) HepG2 cell treated with different doses of AT, PS and joint group (IC_10_, IC_25_ and IC_50_) for 24 h and then stained with MitoHealth stain. (**J**) HepG2 cell treated with different doses of AT, PS and joint group (IC_10_, IC_25_ and IC_50_) for 24 h and then stained with Image-iT^®^ DEAD Green™ viability stain. (**K**) The expression of cytochrome c in HepG2 cells. The cells were treated with high-dose separate PS or AT and joint group for 24 h. (**L**) Quantitation of cleaved-caspase-3 protein expression by densitometry. The data were normalized by using the GAPDH signal. The data are presented as mean ± SD. * and ** indicate *P* < 0.05 and *P* < 0.01, respectively, compared with the control group; ^#^ and ^##^ indicate *P* < 0.05 and *P* < 0.01, respectively, compared with AT and PS group.

The changes of MMP and CMP were evaluated in HepG2 cells after 24 h of AT and PS exposure by using HCS Mitochondrial Health Kit. As shown in Figure 3, after exposure to AT and PS, compared with the control group, the MMP decreased significantly in a dose-dependent manner, indicating that drug-induced toxicity led to cell damage. The phenomenon of CMP changes only occurred in the middle and high-dose joint groups (*P* < 0.05). Furthermore, the protein levels of cytochrome c increased after exposure to the additives, and the joint group had a more conspicuous effect (Figure 3K,L). The data indicate that all joint groups showed a synergistic joint action when compared to the separate groups (*P* < 0.05).

### 2.3. AT Together with PS Induced the Release of ROS in HepG2 Cells

**Figure 4:**
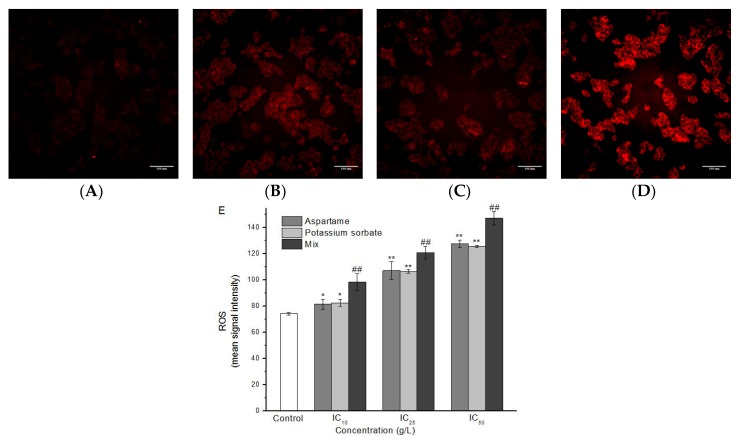
Imaging of ROS in HepG2 cells induced by AT and PS. The MD ImageXpress Micro XLS system was used to obtain images of HepG2 cells. (**A**) ROS accumulation of HepG2 cells in control group for 24 h. (**B**) ROS accumulation of HepG2 cells in AT group for 24 h. (**C**) ROS accumulation of HepG2 cells in PS group for 24 h. (**D**) ROS accumulation of HepG2 cells in joint group for 24 h. Scale bars = 100 μm. (**E**) HepG2 cell treated with different doses of AT, PS and joint group (IC_10_, IC_25_ and IC_50_) at 24 h and then stained with CellROX^®^ Deep Red reagent. The data are presented as mean ± SD. * and ** indicate *P* < 0.05 and *P* < 0.01, respectively, compared with the control group; ^#^ and ^##^ indicate *P* < 0.05 and *P* < 0.01, respectively, compared with AT and PS group.

The levels of ROS were evaluated in HepG2 cells after 24 h of AT and PS exposure by using HCS CellROX^®^ Oxidative Stress Reagent. As shown in Figure 4, the levels of ROS in separate AT and PS group were significantly increased compared with the normal control group (*P* < 0.05), and showed a dose-dependent manner. Compared with the separate group, the levels of ROS in the joint group were significantly improved. The additive effect model was evaluated as synergistic, compared to the separate group (*P* < 0.05).

### 2.4. AT Together with PS Induced DNA Damage in HepG2 Cells

**Figure 5:**
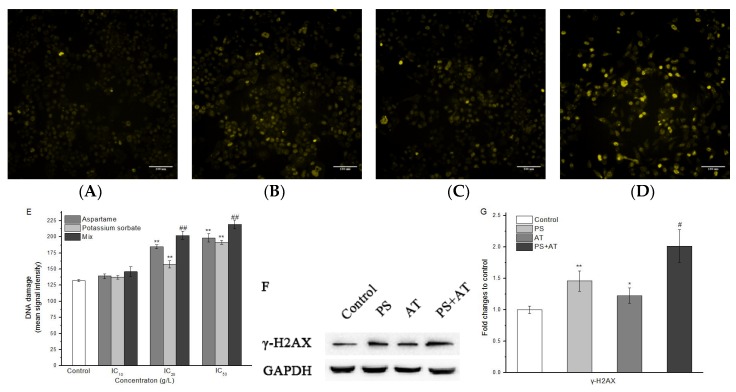
Imaging of AT and PS on DNA damage in HepG2 cells. The MD ImageXpress Micro XLS system was used to obtain images of HepG2 cells. (**A**) DNA damage of HepG2 cells in control group for 24 h. (**B**) DNA damage of HepG2 cells in AT group for 24 h. (**C**) DNA damage of HepG2 cells in PS group for 24 h. (**D**) DNA damage of HepG2 cells in joint group for 24 h. Scale bars = 100 μm. (**E**) HepG2 cell treated with different doses of AT, PS and joint group (IC_10_, IC_25_ and IC_50_) at 24 h and then stained with primary pH2AX antibody solution and secondary anti-mouse IgG antibody solution. (**F**) The expression of γ-H2AX in HepG2 cells. The cells were treated with high-dose separate PS or AT and joint group for 24 h. (**G**) Quantitation of γ-H2AX protein expression by densitometry, and the data were normalized by using the GAPDH signal. The data are presented as mean ± SD. * and ** indicate *P* < 0.05 and *P* < 0.01, respectively, compared with the control group; ^#^ and ^##^ indicate *P* < 0.05 and *P* < 0.01, respectively, compared with AT and PS group.

The phosphorylation of H2A histones was evaluated in HepG2 cells after 24 h of AT and PS exposure by using HCS DNA damage kit. As shown in Figure 5, compared with the control group, only the low-dose group had no effect on increasing the phosphorylation of H2A histones level (*P* > 0.05). Additionally, Western blotting analysis showed that the protein content of γ-H2AX in the joint group was found to be generally higher than that of the separate group (Figure 5F,G). All of the joint groups showed a synergistic joint action compared to the separate groups (*P* < 0.05).

### 2.5. AT Together with PS Induced Apoptosis through Activation of p53, Bax and Bcl-2 in HepG2 Cells

**Figure 6:**
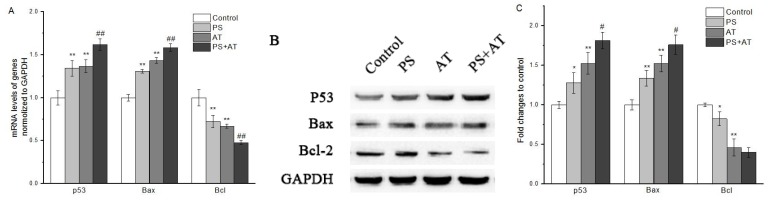
QRT-PCR and Western blot analyses of mRNA and protein expression in HepG2 cells. The p53, Bax and Bcl-2 expression in various groups were analyzed by qPCR (**A**) and Western blot (**B**). (**C**) The protein contents of p53, Bax and Bcl-2 in HepG2 cells exposed to high-dose separate PS or AT and joint group for 24 h. GAPDH mRNA was the internal standard, GAPDH staining was used to ensure loading of the proteins. Quantitation of p53, Bax and Bcl-2 protein expression by densitometry, and the data were normalized by using the GAPDH signal. The data are presented as mean ± SD. * and ** indicate *P* < 0.05 and *P* < 0.01, respectively, compared with the control group; ^#^ and ^##^ indicate *P* < 0.05 and *P* < 0.01, respectively, compared with AT and PS group.

The results of qPCR showed that AT and PS exposure basically promoted p53 transcription. Joint group showed a higher level of transcription of p53 when compared to the separate group (*P* < 0.05) (re 6A). Similarly, Western blot also showed that p53 protein levels in joint group after 24 h of exposure were obviously higher than that of separate group (Figure 5B,C). Meanwhile, our data indicate that joint group could significantly increase the protein level of Bax and decrease the protein level of Bcl-2 (*P* < 0.05) (Figure 6). These results suggest that the activation of p53, Bax and Bcl-2 was required in AT together with PS-induced apoptosis.

## 3. Discussion

The lack of an efficient and accurate way to evaluate the safety of compounds and additives is an important challenge for the cosmetics, pharmaceutical, food and chemical industries. As a class of natural or synthetic compounds, the safety of food additives has been widely concerned. The basis for the present safety evaluations of compounds are toxicity assessments of vitro models and studies in regulatory animals. Although concordance between animal and human toxicity is poor for hypersensitivity and cutaneous reactions, it is much higher for gastrointestinal, hematological and cardiovascular toxicity [12]. Japanese scholars have studied the ability of six kinds of food additives to damage ddY mice, and found that artificial pigments and sweeteners had higher genetic toxicity and higher DNA damage rate [13]. Lau studied the ability of bright blue and l-glutamic acid, and quinoline yellow and aspartame to induce neurotoxicity of NB2a cells. Significant synergy was observed between combinations of Brilliant Blue with l-glutamic acid. Both combinations had a straightforward additive effect on cytotoxicity [14]. Additionally, many studies have revealed the potential genotoxic or mutagenic effects of the additives [15,16,17,18,19,20]. Based on these results, we believe that more extensive assessment of food additives in current use is warranted. In this study, MTT assay indicated that the inhibitory effect of AT and PS on the proliferation of HepG2 cells, which showed a dose–effect relationship. The results in Figure 1 show that both AT and PS were able to induce cytotoxicity in HepG2 cells in a dose-dependent manner with an IC_50_ of 0.48 and 1.25 g/L at 24 h, respectively. Subsequent experiments found that AT group, PS group and joint group led to different levels of apoptosis, showing a time–dose–effect relationship. To further explore the mechanism of cell apoptosis induced by the AT together with PS, the changes of MMP, CMP, ROS, DNA damage, caspase family genes expression, p53, cytochrome c, γ-H2AX, Bax and Bcl-2 protein levels were detected.

Apoptosis is an active cell death in certain physiological or pathological conditions for maintaining the body’s normal physiological processes and functional activities, which is characterized by cell shrinkage, chromatin condensation, nucleus condenses and the formation of apoptotic bodies [21]. In HepG2 cells treated with AT and PS, chromatin of the nucleus’ condensations were observed by Hoechst 33,342 staining (Figure 2B–D). The results also reveal that the rate of apoptosis increased significantly in a dose- and time-dependent manner after exposed to AT and PS (Figure 2F–H,I). Caspase-3 is a downstream effector and common molecule of the apoptosis pathway [22]. Western blot analysis showed that the level of cleaved-caspase-3 increased after exposed to AT and PS (Figure 2J,K). These results suggest that, compared with single AT and PS, the joint group could induce more serious apoptosis in HepG2 cells, and caspase-3 may play an important role in it.

MMP is a marker commonly used in potentially hepatotoxic compounds. Loss of MMP may cause various detrimental consequences, such as energy metabolism, calcium homeostasis, reactive oxygen species production, ATP consumption and cell death [23,24,25]. In addition, the release of certain mitochondrial proteins is essential for the induction and administration of caspase-dependent apoptosis. In mitochondrial pathway, when cells were stimulated, the MMP was changed, which induced cytochrome c to release from mitochondria and led to the activation of caspase-9, which then activated downstream caspases such as caspase-3, giving rise to apoptosis [26]. In this study, compared with the separate group, joint group could decrease the MMP more obviously, accompanied by the increase of CMP (Figure 3). Meanwhile, the protein expression of cytochrome c was increased markedly after treatment with AT, PS and joint group (Figure 3K,L). Both AT and PS showed a synergistic effect on MMP when compared to the separate groups (*P* < 0.05). The Bcl-2 family played an important role in the apoptosis of the apoptotic proteins. Many apoptotic stimuli induce mitochondria-mediated apoptosis in cancer cells by downregulation of Bcl-2/Bax ratio, which is anti-apoptotic and/or up-regulation of Bax/Bad/Bid, which are pro-apoptotic [27,28]. Our data show that AT and PS significantly decreased the ratio of Bcl-2/Bax in a dose-dependent manner, while the joint group had a more pronounced effect (*P* < 0.05) (Figure 6). Taken together, these results indicate that the AT together with PS-induced apoptosis in HepG2 cells was caspase-dependent. Furthermore, mitochondrial pathway may be involved in AT together with PS-induced apoptosis.

Oxidative stress is an important hepatotoxicity mechanism. It is produced by an imbalance between ROS formation in the cell and the activity of its antioxidant systems [29]. An excess of ROS can induce oxidation and damage to DNA, lipids, proteins, and other macromolecules, which may lead to apoptosis [30]. In this study, intracellular ROS levels in separate AT and PS groups were significantly increased. Besides, the levels of ROS in the joint group were higher than those of the separate groups (Figure 4). Both AT and PS showed a synergistic effect on ROS when compared to the separate groups (*P* < 0.05). This result suggested that AT together with PS may significantly cause oxidative stress to the cells and ROS may be involved in AT together with PS-induced apoptosis (*P* < 0.05).

Double strand breaks in chromosomal DNA are potentially lethal lesions in mammalian cells. One of the known reactions to double strand break formation is the phosphorylation of H2A histones. p53 plays an important role in the inhibition of cell growth and the induction of apoptosis after DNA damage [31,32,33]. p53 genes were activated after the DNA damage, and promoted the expression of Bax and the low expression of anti-apoptotic gene Bcl-2, finally activating caspase-3, initiating caspase cascade reaction and resulting in apoptosis. In this study, compared with the separate group, the middle and high-dose joint groups had effects on increasing the phosphorylation of H2A histones level (re 5). Meanwhile, the protein expression of p53 was increased after treatment with AT and PS, and the changes of joint group were more marked (Figure 6). Both AT and PS showed a synergistic effect on DNA damage when compared to the separate group (*P* < 0.05). The data indicate that AT together with PS may cause DNA damage to the cells and p53 may play an important role in AT together with PS-induced apoptosis.

**Figure 7:**
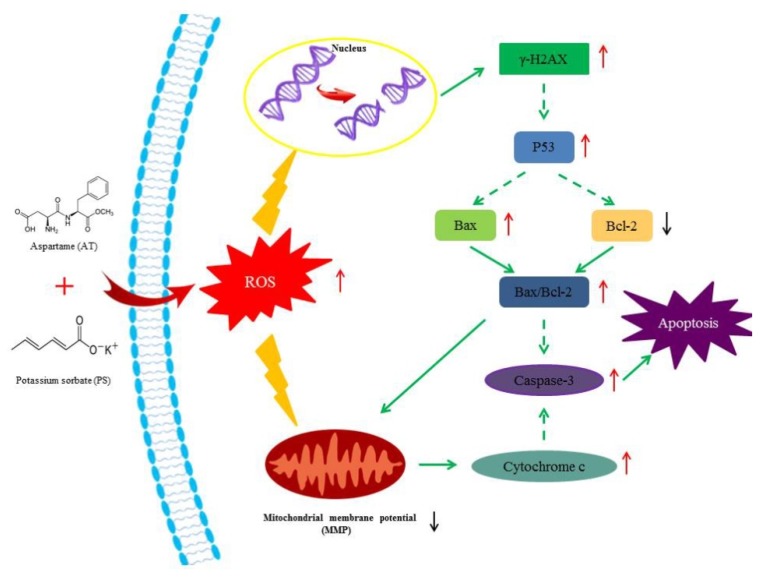
Suppositional pathway of AT together with PS-induced apoptosis in HepG2 cells. AT together with PS induced mitochondrial signaling pathway-mediated apoptosis in HepG2 cells. ROS was also involved in this pathway. Short upward arrows symbolize the up-regulated mRNA or protein content, while short downward arrows symbolize the down-regulated mRNA or protein content. Solid lines symbolize direct action, while dotted lines symbolize possible actions.

In summary, the present study revealed that AT and PS could induce apoptosis in HepG2 cells, and the joint group had a deeper effect. Apoptosis is the main biochemical and molecular mechanism for inhibiting cell proliferation. Both AT and PS had synergistic effects on MMP, ROS and DNA damage. Moreover, excessive ROS induced by AT together with PS was a key initiating factor for the apoptosis and the mechanism of mediation apoptosis may be connected with the mitochondrial pathway in which p53 may play an important role, as shown in Figure 7. To further explore the specificity of joint effect of AT and PS, different types of cells could be compared.

## 4. Materials and Methods

### 4.1. Cell Culture and Treatment

HepG2 cells (Shanghai Cell Bank, Chinese Academy of Sciences, Shanghai, China) were grown at 37 °C, 5% CO_2_ in DMEM, supplemented with 10% (*v*/*v*) Thiazolyl Blue Tetrazolium Bromide (FBS) (HyClone, Logan, UT, USA), streptomycin (100 mg/mL), and penicillin (100 U/mL). At 80% confluence, cells were harvested by trypsinization (0.5% trypsin/2.6mM EDTA) (HyClone, USA), and subcultured according to experimental requirements. The appropriate amounts of potassium sorbate (PS) (Ryon, Shanghai, China) and aspartame (AT) (Nutrasweet, Saint Louis, MI, USA) were dissolved in DMEM culture medium (HyClone, Logan, UT, USA) and filtered through 0.22 μm filter, corresponding to the final concentrations of 0–2.50 g/L and 0–1.00 g/L, respectively.

### 4.2. Cell Viability Assay

Cell viability and cytotoxicity were determined by using the Thiazolyl Blue Tetrazolium Bromide (MTT) assay. The HepG2 cells were seeded in 96-well plates with 5 × 10^3^ cells/well in a complete growth medium for 24 h. Cells were further treated with or without PS and AT and incubated for 24 h. Then, the cell viability was determined according to the instruction of a commercial MTT kit (Beyotime, Nantong, China). After incubation, cells were washed twice with PBS and incubated with MTT solution (1 mg/mL) for 4 h at 37 °C. The resulting formazan crystals were dissolved in 150 mL DMSO and the optical density was measured at 490 nm, using a microplate reader (Thermo Scientific, Waltham, MA, USA). The inhibition rate (%) was calculated by the following formula: (OD 490 (control) − OD 490 (treatment))/OD 490 (control) × 100, where OD 490 (treatment) is the mean OD value of cells treated with the various PS and AT, and OD 490 (control) is the mean OD value of untreated cells. 

### 4.3. Evaluation of Joint Effect

In this study, the effect method―”effect additivity”—was used to evaluate the joint effect mode of food additives [14]. It can be described by the formula M = E(A/2 + B/2)/E(A), which assumes that there is no interaction between the substances in the mixture, where E(A/2 + B/2) refers to the effect of the combination of half dose of A and B, E(A) and E(B) are the effects of A and B, respectively, and E(A) = E(B). If there is no significant difference, it is additive effect; and if there is significant difference, it is the synergistic effect when M is greater than 1, and antagonistic effect when M is less than 1. If the dose is mixed with the IC_25_ of each compound, the expected mortality rate should be 50%. The mixed mortality and expected mortality are compared to evaluate the combined toxicity of the mixture. In the separate group, IC_10_ was used as the low-dose group, IC_25_ as the middle-dose group and IC_50_ as the high-dose group. The concentrations of PS and AT were 0.47, 0.77, and 1.25 g/L and 0.21, 0.32, and 0.48 g/L, respectively, not exceeding the ADI. In the joint group, PS IC_5_ + AT IC_5_ was used as the low-dose joint group, PS IC_12.5_ + AT IC_12.5_ as the middle-dose joint group and PS IC_25_ + AT IC_25_ as the high-dose joint group. The concentrations of the joint groups were 0.34 + 0.16, 0.53 + 0.23, and 0.77 + 0.32 g/L, respectively. QRT-PCR and Western blot were performed in a high-dose of separate group and joint group.

### 4.4. Cell Apoptosis Detection

HepG2 cells were seeded in 96-well plates with 1 × 10^4^ cells/well in a complete growth medium for 24 h. Cells were further treated with or without PS and AT and incubated for 12, 24, and 48 h to determine the actuation duration, and 24 h was chosen was the final action time. HepG2 cells were double-stained with Hoechst 33,342 (10 μg/mL) and PI (10 μg/mL) and then incubated at room temperature for 10 min. HCA was used for imaging and analysis.

### 4.5. Mitochondrial Health Detection

Mitochondrial health kit (Thermo Scientific, USA) was used to measure the changes in MMP. HepG2 cells were seeded in 96-well plates with 5 × 10^3^ cells/well in a complete growth medium for 24 h. Cells were further treated with or without PS and AT and incubated for 24 h. According to the kit instructions to join the right amount of fluorescent dye, cells were stained with Hoechst 33,342 and MitoHealth stain. The Image-iT^®^ DEAD Green™ (Thermo Scientific, Waltham, MA, USA)-viability stain was used to dye DNA to characterize the changes of CMP. HCA was used for imaging and analysis.

### 4.6. Measurement of Intracellular ROS Release

Cellular ROS was measured with HCS CellROX^®^ Oxidative Stress reagents (Thermo Scientific, USA). HepG2 cells were seeded in 96-well plates with 5 × 10^3^ cells/well in a complete growth medium for 24 h. Cells were further treated with or without PS and AT and incubated for 24 h, and then stained with HCS CellROX^®^ Deep Red reagent according to the kit instructions. HCA was used for imaging and analysis.

### 4.7. DNA Damage Detection

HCS DNA Damage Kit (Thermo Scientific, USA) was used to characterize the genetic damage marker-phosphorylated histone H2AX. HepG2 cells were seeded in 96-well plates with 5 × 10^3^ cells/well in a complete growth medium for 24 h. Cells were further treated with or without PS and AT and incubated for 24 h, and then stained with primary pH2AX antibody solution and secondary anti-mouse IgG antibody solution according to the kit instructions. HCA was used for imaging and analysis.

### 4.8. RNA Extraction and Quantitative Real-Time RT-PCR

After 24 h of PS and AT exposure, the cells were washed twice with PBS, recollected, and stored at −80 °C for the following RNA isolation. The total RNA was isolated from the cells by using Trizol (Applied Biosystems, Waltham, MA, USA) method. Nanodrop spectrophotometer (Thermo Scientific, USA) was used to determine the purity and concentration of RNA before reverse transcription. The cDNA was synthesized by reverse transcription of total RNA by using the High-Capacity cDNA Reverse Transcription Kits (Applied Biosystems, USA). The expressions of p53, cytochrome c, Bax, Bcl-2, and caspase-3 at mRNA level were determined by qRT-PCR using the PowerUp^TM^ SYBR^TM^Green Master Mix (Applied Biosystems, USA). The amount of target mRNA was normalized to GAPDH mRNA (an internal control) and was determined using the formula 2^−△△Ct^ [34]. The primers were designed according to the gene sequences of human GAPDH, p53, Bax, and Bcl-2 obtained from GenBank. GAPDH primers were: forward: 5′-CGGAGTCAACGGATTTGGTCGTAT-3′ and reverse: 5′-AGCCTTCTCCATGGTGG TGAAGAC-3′. P53 primers were: forward: 5′-TAGTGTGGTGGTGCCCTATG-3′ and reverse: 5′-CCAGTGTGATGATGGTGAGG-3′. Bax primers were: forward: 5′- ATGG AGGGGTCCGGGGAG-3′ and reverse: 5′-TGGAAGAAGATGGGCTGA-3′. Bcl-2 primers were: forward: 5′-ATGTGTGTGGAGAGCGTCAA-3′ and reverse: 5′- GAGACAGCC AGGAGAAATCAA-3′.

### 4.9. Western Blot Analysis

After incubation with AT and PS for 24 h, the cells were washed twice with ice-cold PBS, harvested and then lysed with ice-cold protein extraction buffer (Beyotime, China). The total cellular protein concentration was determined by using the protein assay kit (Beyotime, China). The protein sample was mixed with an equal volume of 4× protein loading buffer, boiled for 5 min and stored at −20 °C. Equal protein amounts were separated by using a 12% sodium dodecyl sulfate-polyacrylamide gel electrophoresis (SDS-PAGE) and finally transferred to a polyvinylidene fluoride membrane (PVDF membranes). The membrane was blocked using 5% skimmed milk for 2 h at 25 °C, and then immunoblotted overnight at 4 °C with primary antibodies p53 (1:1000 dilution), cytochrome c (1:1000 dilution), γ-H2AX(1:1000 dilution), caspase-3(1:1000 dilution), Bax (1:1000 dilution), Bcl-2 (1:1000 dilution) and GAPDH (1:1000 dilution) (CST, USA), respectively. After washing three times with TBST for 10 min each, the membrane was incubated in the anti-rabbit IgG, HRP-linked antibody (1:5000 dilution) (CST, USA), at 25 °C for 1 h. Then, the membrane was washed again with TBST for three times and the signal was detected by ECL Plus Kit (Applied Biosystems, USA). Molecular mass of bands was verified using a Multicolor Protein Marker (Beyotime, China). GAPDH protein was used as an endogenous control in this experiment. Quantity one was used for gray scale value calculation of Western blot images. 

### 4.10. Statistical Analysis

All data were expressed as mean ± SD of three independent experiments and analyzed using one-way analysis of variance followed by the least significant difference determination with SPSS 19.0 software. *P* < 0.05 or *P* < 0.01 was considered statistically significant.
